# Hepatocyte-Conditional Knockout of Phosphatidylethanolamine Binding Protein 4 Aggravated LPS/D-GalN-Induced Acute Liver Injury *via* the TLR4/NF-κB Pathway

**DOI:** 10.3389/fimmu.2022.901566

**Published:** 2022-07-08

**Authors:** Xiao-qin Qu, Qiong-feng Chen, Qiao-qing Shi, Qian-qian Luo, Shuang-yan Zheng, Yan-hong Li, Liang-yu Bai, Shuai Gan, Xiao-yan Zhou

**Affiliations:** ^1^ Department of Pathophysiology, Medical College of Nanchang University, Nanchang, China; ^2^ Department of Pathology, Medical College of Nanchang University, Nanchang, China; ^3^ The Center of Laboratory Animal Science, Nanchang University, Nanchang, China; ^4^ Department of Forensic Medicine, Medical College of Nanchang University, Nanchang, China; ^5^ The Second Clinical Medical College, Nanchang University, Nanchang, China; ^6^ Jiangxi Province Key Laboratory of Tumor Etiology and Molecular Pathology, Medical College of Nanchang University, Nanchang, China

**Keywords:** PEBP4, acute liver injury, TLR4, NF-κB, inflammation, apoptosis

## Abstract

Acute liver injury (ALI) is a disease that seriously threatens human health and life, and a dysregulated inflammation response is one of the main mechanisms of ALI induced by various factors. Phosphatidylethanolamine binding protein 4 (PEBP4) is a secreted protein with multiple biological functions. At present, studies on PEBP4 exist mainly in the field of tumors and rarely in inflammation. This study aimed to explore the potential roles and mechanisms of PEBP4 on lipopolysaccharide (LPS)/D-galactosamine (D-GalN)**-**induced ALI. PEBP4 was downregulated after treatment with LPS/D-GalN in wild-type mice. PEBP4 hepatocyte-conditional knockout (CKO) aggravated liver damage and repressed liver functions, including hepatocellular edema, red blood cell infiltration, and increased aspartate aminotransferase (AST)/alanine aminotrans-ferase (ALT) activities. The inflammatory response was promoted through increased neutrophil infiltration, myeloperoxidase (MPO) activities, and cytokine secretions (interleukin-1β, IL-1β; tumor necrosis factor alpha, TNF-α; and cyclooxygenase-2, COX-2) in PEBP4 CKO mice. PEBP4 CKO also induced an apoptotic effect, including increasing the degree of apoptotic hepatocytes, the expressions and activities of caspases, and pro-apoptotic factor Bax while decreasing anti-apoptotic factor Bcl-2. Furthermore, the data demonstrated the levels of Toll-like receptor 4 (TLR4), phosphorylation-inhibitor of nuclear factor kappaB Alpha (p-IκB-α), and nuclear factor kappaB (NF-κB) p65 were upregulated, while the expressions of cytoplasmic IκB-α and NF-κB p65 were downregulated after PEBP4 CKO. More importantly, both the NF-κB inhibitor (Ammonium pyrrolidinedithiocarbamate, PDTC) and a small-molecule inhibitor of TLR4 (TAK-242) could inhibit TLR4/NF-κB signaling activation and reverse the effects of PEBP4 CKO. In summary, the data suggested that hepatocyte-conditional knockout of PEBP4 aggravated LPS/D-GalN-induced ALI, and the effect is partly mediated by activation of the TLR4/NF-κB signaling pathway.

## Introduction

Acute liver injury (ALI) is a disease characterized by the destruction of liver defense function and the induction of uncontrolled inflammation (1). Following severe or persistent liver injury will ultimately provoke acute liver failure (ALF). Once ALI or ALF occurs, the affected individual’s life and health become seriously threatened (2, 3). So far, no specific drugs for ALI or ALF exist for use as clinical treatments. Therefore, it is vital to explore specific intervention targets and design more effective therapeutic drugs.

Inflammation and apoptosis are the main pathological manifestations of ALI (4, 5). The injection of LPS/D-GalN can induce the most classic and common model of ALI (6–8). The TLR4/NF-κB signaling pathway plays the most important role in the inflammatory response (9–11). TLR4, a member of the Toll-like protein family, could bind to LPS on the cell membrane, then activate NF-κB (12). The activation of NF-κB promotes the expression of pro-inflammatory cytokines and further aggravates the inflammatory process (13). Moreover, NF-κB can also regulate apoptosis-related hydrolytic protein caspases along with apoptosis-related genes, such as Bax and Bcl-2 (14, 15). Uncontrollable inflammatory and apoptotic responses ultimately lead to liver injury (16, 17).

PEBP4, a member of the PEBP family, is a secreted protein widely expressed in multiple organs of the human body (18). At present, studies on PEBP4 have mostly focused on cancers (19–23). In addition, members of the PEBP family, such as Raf kinase inhibitor protein (RKIP) and PEBP1, can regulate microglia inflammation by inhibiting the NF-κB signaling pathway (24, 25), and RKIP is known to be closely associated with ALF (26). Furthermore, PEBP4 can also regulate the apoptotic process (27, 28). Based on these prior studies, we speculated about PEBP4 may exacerbate liver injury by facilitating the inflammation release and apoptosis, and the effect was achieved through TLR4/NF-κB signaling pathway. To explore the effects and mechanism of PEBP4 in ALI hepatocyte-conditional knockout of PEBP4 mice were established, and the mouse model of ALI was built *via* the administration of D-GalN combined with LPS. Our research is the first to focus PEBP4 on the field of inflammatory diseases, which will provide a new experimental basis for clinical treatment.

## Materials and Methods

### Reagents

LPS (Escherichia coli, O55: B5), D-GalN and PDTC were obtained from Sigma-Aldrich (St. Louis, MO, USA). TAK-242 was acquired from Abmole Bioscience Inc (Houston, TX, USA). Kits for the detection of AST, ALT and MPO detection kits were purchased from Nanjing Jiancheng Bioengineering Institute (Nanjing, China). Enzyme-linked immunosorbent assay (ELISA) kits for IL-1β and TNF-α were provided by Invitrogen (Carlsbad, CA, USA). Trizol reagents, EasyScript One-Step gDNA Removal, and cDNA Synthesis Super Mix, and PerfectStart Green qPCR Super Mix were produced by TransGen Biotech (Beijing, China). Kits for the detection of caspase-3 and -9 activity came from Abcam (Cambridge, England). A caspase-8 activity detection kit was sourced from BioVision (San Francisco, CA, USA). A transferase-mediated dUTP-biotin nick end labeling assay (TUNEL) kit was acquired from Servicebio Biotech (Wuhan, China). PEBP4 antibody was obtained from RayBiotech (Atlanta, USA). The antibodies of IκB-α and p-IκB-α were produced by Cell Signaling Technology (Boston, MA, USA). Finally, the antibodies of NF-κB p65; caspases-3, -8, and -9; lamin B1; ATP1A1; and GAPDH were provided by Proteintech (Chicago, IL, USA).

### Animals and ALI Model

C57BL/6N WT mice were purchased from Jiangsu Jicui Yaokang Biotechnology Co., Ltd (Jiangsu, China). *PEBP4^flox/+^* mice, and Alb-Cre^+^ mice were supplied by Saiye Biotechnology Co., Ltd (Jiangsu, China) (The loxP sequences with the *PEBP4* gene were introduced by CRISPR/Cas9 technology to allow for the conditional deletion of exon 3, which would result in a null allele upon Cre recombinase-mediated excision. Mutant PEBP4-floxed offspring were generated on a C57BL/6N background and identified by genotyping. All mice were maintained under 25°C and 12h day/night rhythm with sufficient food and sterile water, and the research was approved by the Animal Protection Committee of Jiangxi Medical College of Nanchang University.

After one week of adaptive feeding, *PEBP4^flox/flox^* mice were obtained from the offspring of male and female mice with the *PEBP4^flox/+^* gene, and Alb-Cre^+^ mice were intercrossed with *PEBP4^flox/flox^* mice to obtain *PEBP4^flox/+;Alb-Cre+^* mice. The *PEBP4^flox/flox;Alb-Cre+^* (PEBP4 CKO) mice were generated by breeding male and female *PEBP4^flox/+;Alb-Cre+^* mice together. Finally, PEBP4 CKO mice were used in this experiment. The genotype was identified by polymerase chain reaction (PCR). Sequences of forward and reverse primers are list in the **Table 1**. When enough WT and PEBP4 CKO mice were obtained, each type of mouse was randomly divided into the following four groups(n=12 per group): (1) a control group, which received an intraperitoneal injection with normal saline, (2) an LPS/D-GalN group, which received an intraperitoneal injection with 5 μg/kg of LPS and 300 mg/kg of D-GalN, (3) an LPS/D-GalN+PDTC (NF-κB inhibitor) group, which received 60 mg/kg of PDTC by intraperitoneal injection 30 min prior to receiving 5 μg/kg of LPS and 300 mg/kg of D-GalN; and (4) an LPS/D-GalN+TAK-242 (TLR4 inhibitor) group, which received 3 mg/kg of TAK-242 by intraperitoneal injection 30 min prior to receiving 5 μg/kg of LPS and 300 mg/kg of D-GalN. Half of the mice in each group were sacrificed at 6 h, and their blood and liver tissues were collected (10); the other half were sacrificed at 1.5 h, and their serum was harvested for TNF-α detection.

**Table 1:** Sequence used for PCR.

No	Primer name	Primer sequence (5’-3’)	Band size
1	loxp F	GATCCTGGAGCTACTGAAAGCACTGAG	Flox=251 bp WT=188 bp
	loxp R	GCTATTTACACCACCATGCCCTGC	
2	Alb-Cre F	GAAGCAGAAGCTTAGGAAGATGG	Alb-Cre=390 bp
	Alb-Cre R	TTGGCCCCTTACCATAACTG	
3	PEBP4 dellele F	GATCCTGGAGCTACTGAAAGCACTGAG	PEBP4 KO=277 bp
	PEBP4 dellele R	ACAACCAGAAGGATGAAATCGGAAAC	

### Hematoxylin and Eosin (H&E) Staining

H&E staining was performed according to Yan at el. (7). In a word, liver tissue blocks (approximately 0.5 × 0.5 cm size) were selected at the same position on the right lobe, then steeped in 4% paraformaldehyde for fixation for 24 h. The 4-6μm tissue sections were stained with H&E using a common protocol. To evaluate the degree of acute liver injury, each sample was independently observed by 3 pathologists under a light microscope (Olympus Corporation, Tokyo, Japan) and made an injury score according to hepatocellular edema, red blood cell infiltration, neutrophil infiltration, and liver structural disorders.

### Western Blotting

Western blot was performed according to the standard protocol. Briefly, the total protein, membrane protein, and nuclear protein from tissues were extracted according to the protein extraction kit instructions, then the protein concentrations were detected with a BCA kit (Solarbio, China). Protein extracts (40μg) were fractionated on 12% polyacrylamide-sodium dodecyl sulfate gel, then transferred to nitrocellulose membranes. Non-specific binding to the membrane was blocked by incubation in 5% (w/v) fat-free milk in TBST buffer, followed by incubation with a rabbit or mouse primary polyclonal antibody (1:1000, except for PEBP4 at 1:750) at 4°C overnight. The next day, the membranes were treated with horseradish peroxidase-conjugated goat anti-rabbit or goat anti-mouse secondary antibody (1:5000). Finally, the protein expressions were observed in a gel imaging system (BioRad Laboratories, Hercules, CA, USA) and performed quantitative analysis using the ImageJ software (U.S. National Institutes of Health, Bethesda, MD, USA).

### Real-Time Quantitative PCR Analysis

Total RNA was extracted from liver tissue using Trizol reagents according to the manufacturer’s instructions. The expressions of gene messenger RNA were measured by real-time PCR using the PerfectStart Green qPCR Master Mix. The gene expressions were calculated by using the comparative 2^−ΔΔCT^ method and GAPDH as a reference gene. The primer sequences of GAPDH (as the reference gene) and candidate genes were listed in **Table 2**.

**Table 2:** The primers sequence used for real-time quantitative PCR.

Name	Primer
GAPDH	F:5’-AGGTCGGTGTGAACGGATTTG-3' R:5’-TGTAGACCATGTAGTTGAGGTCA-3’
Bcl-2	F:5’-CCGGGAGAACAGGGTATGATAA-3’ R:5’-CCCACTCGTAGCCCCTCTG-3’
Bax	F:5’-AGGATGCGTCCACCAAGAAGCT-3’ R:5’-TCCGTGTCCACGTCAGCAATCA-3’

### ALT/AST Measurement

After the mice were sacrificed, the blood was placed on ice for 1 h, and then the supernatant was acquired by a cryogenic ultracentrifuge run at 3,000 rpm, 4°C for 10 min. The supernatant was used to detect the activity of ALT and AST according to the manufacturer’s recommendations.

### MPO Activity Measurement

100 mg of liver tissues were accurately weighed out, added a homogenate medium at a weight-to-volume ratio of 1:19, and obtained 5% tissue homogenate. MPO activities were measured by following the instructions of the activity detection kits.

### ELISA

According to the manufacturer’s instructions, standards were serially diluted at 1:1 dilution and the serum was diluted 10-fold by the sample diluent; then 100 µL of various concentrations of standards (0-2000 pg/mL) and serum were pipetted into the 96-well ELISA plate in duplicate. The plates were incubated at room temperature (18°C-25°C) for 2 h with 50 μL of biotin-conjugate. After that, the microwell strips were washed 6 times with approximately 400 μL of wash buffer per well, then added 100 μL of diluted streptavidin-horseradish peroxidase for 1 h at room temperature. Then, the plates were washed again and treated them with 100 μL of TMB substrate solution for 30 min without intense light. The enzymatic reaction was terminated with 100 μL of stop solution, and the absorbance at 450 nm was recorded with a microplate reader (Molecular Devices, American).

### TUNEL

TUNEL was performed using an apoptosis detection kit according to the manufacturer’s instruction. First, the paraffin sections were placed in xylene and gradient alcohol for dehydration, and washed in distilled water. Secondly, proteinase K repair solution was dropped for antigen retrieval and then 0.1% Triton for permeabilization. Subsequently, reaction solution (TDT enzyme:dUTP:buffer, 1:5:50) was added and incubated sections for 2h at 37°C. Finally, nuclei ware stained with DAPI and coverslip covered sections with anti-fade mounting medium. All images were acquired using a fluorescence microscope (Olympus, Japan).

### Measurement of Caspase-3, -8, and -9 Activities

In according with the kit instructions, the kits were taken out of the refrigerator at -20°C and slowly melted. At the same time, the liver tissues were taken out of the refrigerator at -80°C, 400 μL of pre-cooled cell lysate was added and repeatedly ground, and then placed in a 4°C centrifuge at 10,000 g for 1 min. Subsequently, the supernatant was acquired, and the protein concentrations was immediately detected. Next, 50 μL (50-200 μg) sample or 2× reaction buffers was added to the well, respectively, and 50 μL of reaction mix and 5 μL of DEVD-p-NA substrate were added successively. The mixture was shook horizontally and then placed in a constant-temperature water bath at 37°C for 2 h. Finally, the OD value of each well was detected with a microplate reader (Molecular Devices, American) at a wavelength of 405 nm.

### Statistical Analysis

All data were expressed as mean ± standard deviation (SD) values. GraphPad Prism version 8.0 (GraphPad Software, San Diego, CA, USA) was used for mapping, and the results were statistically analyzed by one-way analysis of variance using SPSS version 26.0 (IBM Corporation, Armonk, NY, USA). *P* < 0.05 indicated that the difference was statistically significant.

## Results

### Constructed and Identified the PEBP4 Hepatocyte-Conditional Knockout (CKO) Mice

**Figure 1:**
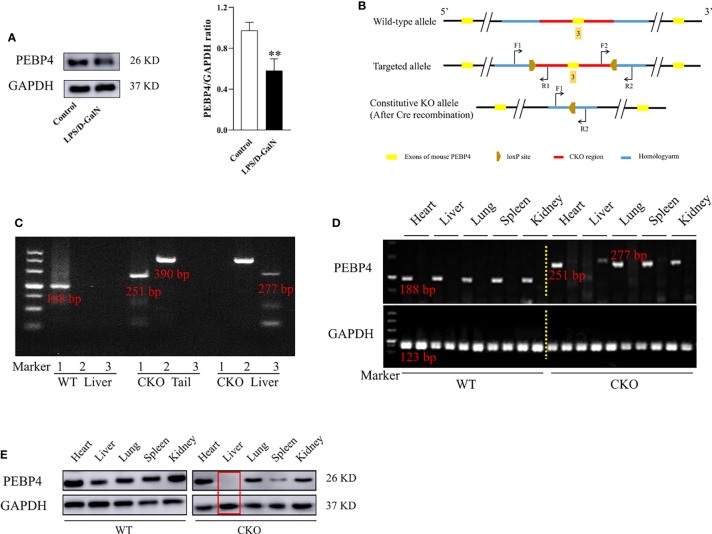
PEBP4 hepatocyte-conditional knockout (CKO) mice were constructed and identified. **(A)**: Western blotting was used to detect the expression of PEBP4. **(B)**: The undisturbed *PEBP4* gene in WT mice (top panel), schematic of loxP sites introduced into exon 3 of the PEBP4 locus for the generation of *PEBP4^flox/flox^* mice (middle panel), and schematic for the generation of PEBP4CKO mice (bottom panel). **(C)**: The LoxP sites flanking the PEBP4 gene, the Cre transgene and PEBP4 allele were detected by PCR analysis of tail DNA and liver DNA. **(D)**: Agarose gel electrophoresis was employed to examine the gene expression of PEBP4 in the heart, liver, lungs, spleen, and kidneys of WT and CKO mice. **(E)**: PEBP4 protein expression was tested by western blotting in the heart, liver, lungs, spleen, and kidneys of WT and CKO mice. Data are presented as mean ± SD values (n = 3). ^**^ *P* < 0.01 compared to WT mice in the control group.

To explore the relationship of PEBP4 with ALI, LPS/D-GalN was used to establish an ALI model among WT mice, and then the protein expressions of PEBP4 were detected by western blotting. The results showed PEBP4 was decreased in ALI (*P* < 0.01, **Figure 1A**). Based on the results of **Figure 1A**, we designed PEBP4 CKO mice to explore the role of PEBP4 in ALI. In this study, we used CRISPR/Cas9 technology to construct *PEBP4^flox/+^* mice, which were maintained with a C57BL/6N background, and then bred *PEBP4^flox/flox^* mice with Alb-Cre mice to obtain PEBP4 CKO mice (**Figure 1B**). Agarose gel electrophoresis and western blotting during the experiment both suggested that the PEBP4 CKO mice were successfully created (**Figures 1C–E**).

### PEBP4 CKO Could Aggravate LPS/D-GalN-Induced ALI

**Figure 2:**
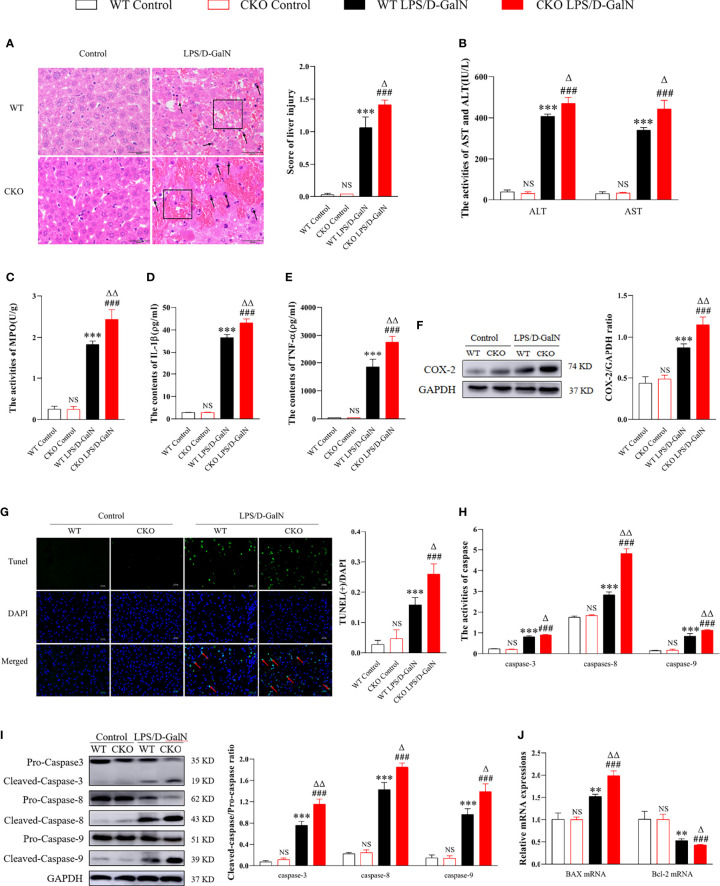
PEBP4 CKO could aggravate LPS/D-GalN–induced ALI. **(A)**: Histological changes of liver tissue (H&E staining, ×400) and the injury scores of HE were quantifies, ↑ for inflammatory cell infiltration, □ for hepatocyte degeneration and red blood cell infiltration. **(B)**: We detected the activities of AST and ALT in serum (n = 6). **(C)**: MPO activities were tested in liver tissue (n = 6). **(D, E)**: The levels of IL-1β and TNF-α in serum were detected by ELISA (n = 6); **(F)**: Western blot was used to examine the expression of COX-2 (n = 3). **(G)**: The apoptotic level of hepatocytes was detected with a TUNEL fluorescence detection kit (the DAPI-stained nuclei were blue and apoptotic nuclei were green, ×400) and positive cells of TUNEL were quantified, ↑ for TUNEL (+) cells. **( H, I)**. The activities and protein expression of caspase-3, -8, and -9 were detected by the activity test kit (n = 6) and western blotting (n = 3), respectively; **(J)**. The mRNA expression levels of Bax and Bcl-2 were detected by real-time PCR. Data are presented as mean ± SD values. ^NS^ *P* > 0.05, ^**^ *P* < 0.01, and ^***^ *P* < 0.001 compared to the WT control group; ^###^ *P* < 0.001 compared to the CKO control group; and ^Δ^ *P* < 0.05 and ^ΔΔ^ *P* < 0.01 compared to the WT LPS/D-GalN group.

According to **Figure 1**, PEBP4 CKO mice were successfully acquired. The liver tissue sections and the results of biochemical kits suggested that there were no significant changes in liver tissue and functions after PEBP4 CKO (^NS^ *P* > 0.05, **Figures 2A, B**). To explore the potential influence of PEBP4 on ALI, the model was induced by injections of LPS/D-GalN in WT mice and PEBP4 CKO mice. Histological changes were detected by H&E staining, and the results showed that, compared with WT mice, LPS/D-GalN aggravated hepatocellular edema, red blood cell infiltration, neutrophil infiltration, and liver structural disorders in the PEBP4 CKO mice (**Figure 2A**). ALT/AST activities were also tested, and data were consistent with the changes in liver pathological damage (**Figure 2B**). Inflammation and apoptosis are two of the most important features in LPS/D-GalN-induced ALI. To assess the degree of the inflammatory response, the levels of MPO, IL-1β, TNF-α, and cyclooxygenase-2 (COX-2) were examined. The results demonstrated the activities of MPO and the expressions of IL-1β, TNF-α, and COX-2 were improved in the LPS/D-GalN groups, although the changes were more obvious in the PEBP4 CKO mice (**Figures 2C–F**). Subsequently, the degree of apoptotic response was evaluated through TUNEL; the expressions and activities of cleaved caspase-3, -8, and -9; and the apoptotic regulatory proteins (Bax and Bcl-2). The results suggested that LPS/D-GalN could induce hepatocyte apoptosis, and a more severe apoptosis response could be observed in PEBP4 CKO mice too (**Figures 2G–I**). Meanwhile, real-time PCR indicated that the expression of Bax was higher and that of Bcl-2 was lower in the PEBP4 CKO mice (**Figure 2J**).

### PEBP4 CKO Activated the TLR4/NF-κB Signaling Pathway

**Figure 3:**
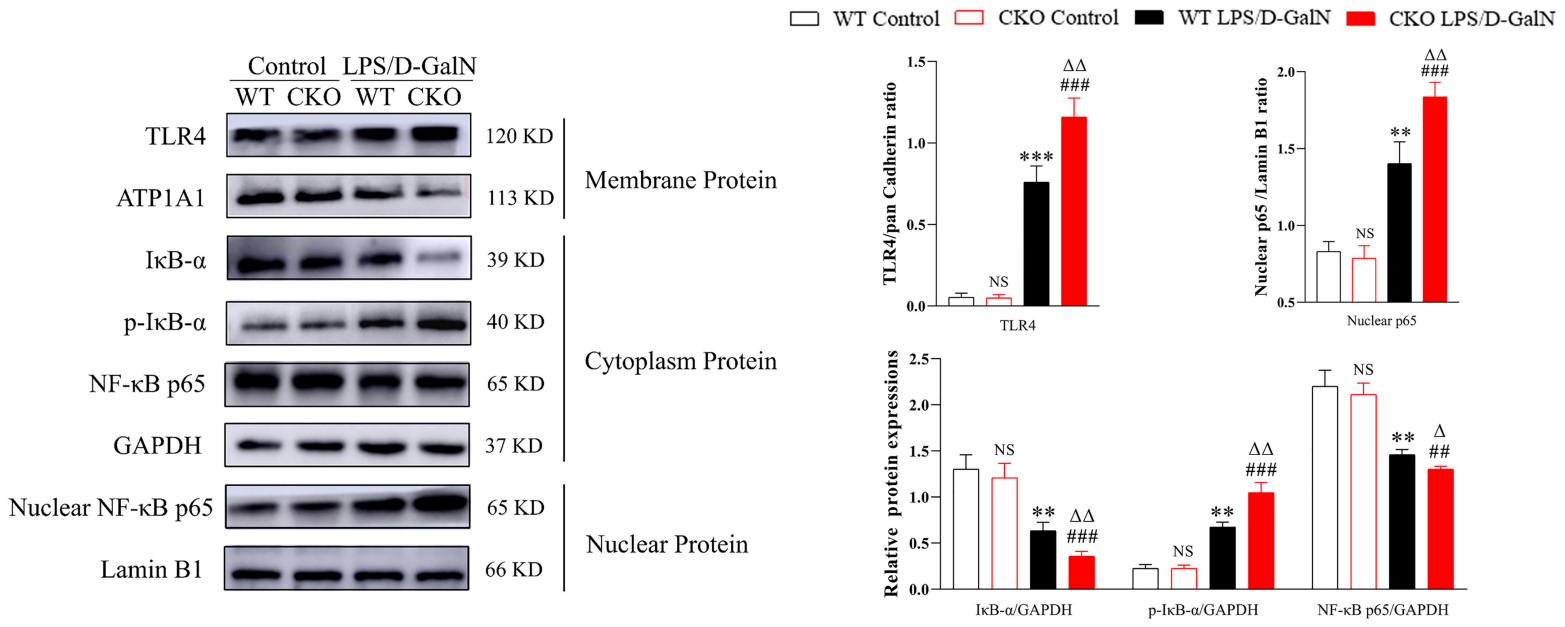
PEBP4 CKO could activate the TLR4/NF-κB signaling pathway. Western blotting was employed to examine the expressions of TLR4, IκB-α, p-IκB-α, nuclear protein NF-κB p65, and cytoplasmic protein NF-κB p65. Data are presented as mean ± SD values (n = 3). ^NS^ *P* > 0.05, ^**^ *P* < 0.01, and ^***^ *P* < 0.001 compared to the WT control group; ^###^ *P* < 0.001 compared to the CKO control group; and ^Δ^ *P* < 0.05 and ^ΔΔ^ *P* < 0.01 compared to the WT LPS/D-GalN group.

The TLR4/NF-κB signaling pathway is one of the important signaling pathways in the regulation of inflammation. In this study, the protein expressions of TLR4, p-IκB-α, and NF-κB p65 were examined. As shown in **Figure 3**, compared to WT mice, LPS/D-GalN could more significantly increase TLR4, p-IκB-α, and nuclear protein NF-κB p65 expressions (*P* < 0.01), and the cytoplasmic protein of IκB-α and NF-κB p65 expression were decreased more obviously in CKO mice (*P* < 0.01). These data indicated that PEBP4 CKO might activate the TLR4/NF-κB signaling pathway.

### TLR4/NF-κB Signaling Pathway Inhibitor Could Partially Reverse the Effects of PEBP4 CKO on LPS/D-GalN-Induced ALI

**Figure 4:**
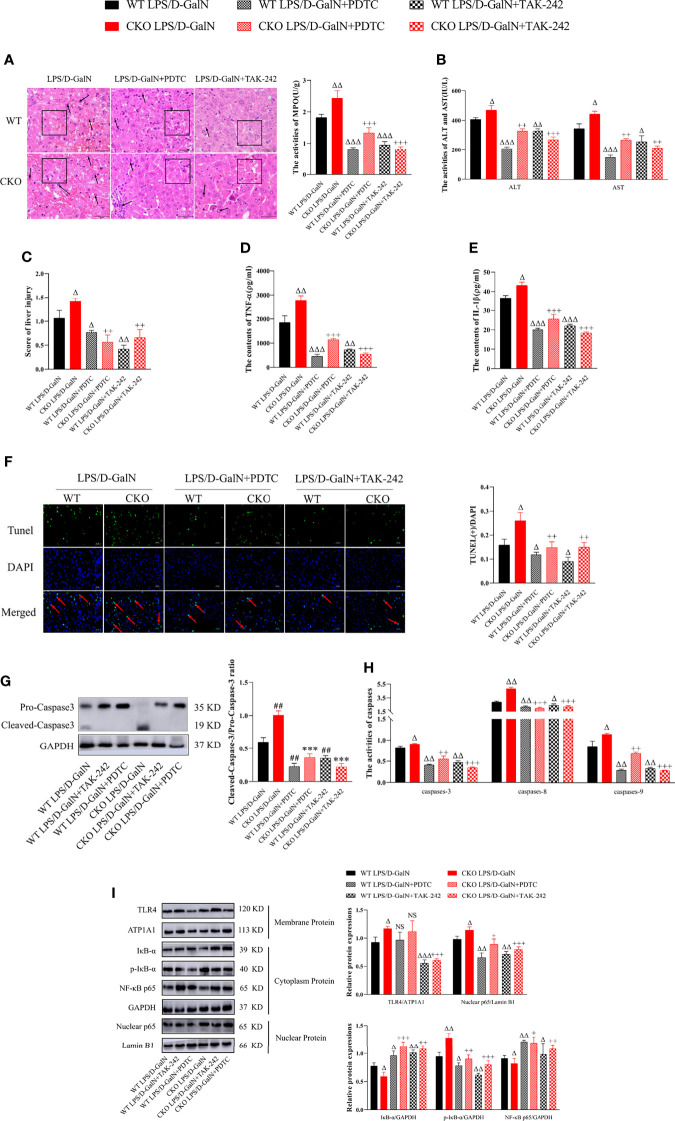
PDTC and TAK-242 could partially counteract TLR4/NF-κB signaling activation induced by PEBP4 CKO in ALI. **(A)**: Histological changes of liver tissue (H&E staining, ×400) and the injury scores of HE was quantifies, ↑ for inflammatory cell infiltration, □ for hepatocyte degeneration and red blood cell infiltration. **(B)**: Serum ALT/AST activities (n = 6). **(C)**: Liver MPO activities (n = 6). **(D, E)**: The contents of serum TNF-α and IL-1β (n = 6). **(F)**: TUNEL staining results of liver tissue (×400) and positive cells of TUNEL were quantified, ↑ for TUNEL (+) cells. **(G)**: The expressions of cleaved caspase-3/pro–caspase-3 (n = 3); **(H)**: The activities of caspase-3, -8, and -9 in liver tissue (n = 6). **(I)**: The expression levels of TLR4, IκB-α, p-IκB-α, NF-κB p65, and nuclear protein NF-κB p65 were detected by western blotting, and the gray levels were analyzed (n = 3). Data are presented as mean ± SD values. ^Δ^ *P* < 0.05, ^ΔΔ^ *P* < 0.01, and ^ΔΔΔ^ *P* < 0.001 compared to the WT LPS/D-GalN group, ^+^ *P* < 0.05, ^++^ *P* < 0.01, and ^+++^ *P* < 0.001 compared to CKO LPS/D-GalN group. NS, no significance.

To further clarify the effects and mechanisms of PEBP4, PDTC and TAK-242 were utilized, and the results showed that both could partially reverse the effects of PEBP4 CKO on ALI, including liver pathological changes (**Figure 4A**); the activities of ALT/AST and MPO (**Figures 4B, C**); the expression levels of inflammatory factors (**Figures 4D, E**); hepatocyte apoptosis (**Figure 4F**); and the expressions and activities of caspases-3, -8, and -9 (**Figures 4G, H**). To provide further evidence regarding the regulatory effect on TLR4/NF-κB signaling, the levels of TLR4, IκB-α, p-IκB-α, and NF-κB p65 were also examined after treatment with PDTC and TAK-242, and the results proved that the phosphorylation and degradation of levels of IκB-α and the nuclear translocation of NF-κB were decreased in the PDTC and TAK-242 intervention groups (**Figure 4I**).

## Discussion

The combined administration of LPS and D-GalN to induce ALI has been widely considered as a model given the pathological changes are similar to endotoxin-induced ALI in humans, and this approach could be utilized to explore the exact pathogenesis of ALI (6, 29). In this study, we first established the model in WT mice and found that PEBP4 expression was significantly inhibited in ALI. The results suggested PEBP4 may be a possible intervention target for ALI. PEBP4 is a secreted protein with a variety of biological functions. Available studies on PEBP4 have mainly focused on cancers, and PEBP4 is highly expressed in cancer tissues (19–23), but the effects of PEBP4 expression on tissue damage have not been reported, which attracted our attention. To investigate the effects of PEBP4 on ALI and the potential mechanisms, PEBP4 CKO mice were established and liver damage was observed after LPS/D-GalN treatment. The results of pathological changes showed that the LPS/D-GalN group of PEBP4 CKO mice had more red blood cell infiltration in liver tissues and more serious hepatocellular edema than that in WT mice. In addition, the activities of ALT and AST were higher in PEBP4 CKO mice. These results indicated that PEBP4 CKO aggravated liver damage and reduced liver function.

The inflammatory response is the major contributing factor in the development of LPS/D-GalN-induced ALI. Various pro-inflammatory cytokines, such as TNF-α and IL-1β, are secreted, and then neutrophil activation and infiltration are elicited by TNF-α, which has been considered an important hinge in the progress of LPS/D-GalN-induced ALI (30–33). The results in this research coincided with this point. In this study, the activity of MPO, which reflected the degree of neutrophil infiltration, and the expressions of inflammatory factors (IL-1β, TNF-α, and COX-2) were all increased in PEBP4 CKO mice. Some studies have reported that PEBP family member PEBP1 and RIPK have anti-inflammatory roles, but little is known about the impact of PEBP4 in inflammation (24, 25). In this experiment, the data demonstrated that, after hepatocyte-conditional knockout of PEBP4, all the inflammatory indicators (pathological changes, MPO activity, IL-1β, TNF-α, and COX-2) were more significantly serious in CKO mice, suggesting PEBP4 CKO could promote inflammation and PEBP4 may be a target to control ALI. On the other hand, hepatocyte apoptosis is a common phenomenon in LPS/D-GalN-induced ALI models, and it is also the cause of liver function decrease and even death. Therefore, controlling apoptosis is also a key target for mitigating liver damage (34, 35). TNF-α initiated the death receptor-dependent apoptosis pathway through its receptors, leading to the activation of the caspase cascade (36–38). The results in this research indicated that PEBP4 CKO increased TNF-α secretion. In addition, it has been reported that PEBP4 could enhance cell resistance to TNF-α-induced apoptosis (39). So, we also speculate that PEBP4 is involved in the regulation of apoptosis in LPS/D-GalN-induced ALI in this investigation. TUNEL staining and the activities and expressions of apoptosis-related factors were examined in this study. The data suggested the number of TUNEL (+) cells in hepatic tissues; the expression levels of cleaved caspase-3, -8, and -9; and the activities of caspase-3, -8, and -9 were increased in PEBP4 CKO mice after treatment with LPS/D-GalN. Meanwhile, the expression of pro-apoptotic Bax was upregulated, while that of anti-apoptotic factor Bcl-2 was downregulated. This suggested that PEBP4 CKO could facilitate LPS/D-GalN-induced hepatocyte apoptosis and PEBP4 may be a target to resist ALI also.

A large number of studies have shown that the TLR4/NF-κB signaling pathway plays an important role in the development of ALI induced by LPS/D-GalN (10, 40, 41). TLR4 is a transmembrane receptor existing in Kupffer cells whose natural ligand is LPS. When TLR4 combines with LPS, the innate immune and inflammatory responses of liver tissue are initiated (42–44). NF-κB, an important transcription factor, could be activated by TLR4, and then NF-κB p65 is transferred from the cytoplasm to the nucleus to regulate the expression of inflammatory cytokines, such as IL-1β, IL-6, TNF-α, caspases, Bax, and Bcl-2 (14, 15, 45, 46). In this study, TLR4/NF-κB signaling pathway-related proteins were detected, and it was found that TLR4 and nuclear NF-κB p65 protein expression increased and IκB-α and cytoplasm NF-κB p65 expression decreased in PEBP4 CKO mice, suggesting that PEBP4 deficiency may activate the TLR4/NF-κB signaling pathway. To further clarify the effects and mechanisms of PEBP4, PDTC and TAK-242 were used. Subsequently, the data hinted that phosphorylation and degradation of IκB were inhibited and the level of NF-κB translocation into the nucleus was reduced. More importantly, the activities of MPO and ALT/AST decreased, the secretion levels of TNF-α and IL-1β reduced, the number of TUNEL (+) cells fell, and the level of cleaved caspase-3 dropped in the two inhibitor groups. All these data suggest that both inhibitors could partially reverse PEBP4 CKO induced activation of the TLR4/NF-κB signaling pathway and then restrain inflammation and apoptosis.

In this study, we found that PEBP4 expression was decreased in ALI and PEBP4 CKO aggravated ALI, contrasting with the increased expressions of PEBP4 in tumor tissues and PEBP4’s promotion of tumor development. The different effects may be mainly related to the expression levels. PEBP4 could have a protective effect at a low or normal expression level. However, once PEBP4 was overexpressed, it would adopt different or even completely opposite biological effects, and the specific mechanisms, including the interaction between PEBP4 and TLR4, need to be further investigated and explored. Furthermore, the defect of this article is that the cell experiment is not completed synchronously, which will be further improved in the later stage.

**Figure 5:**
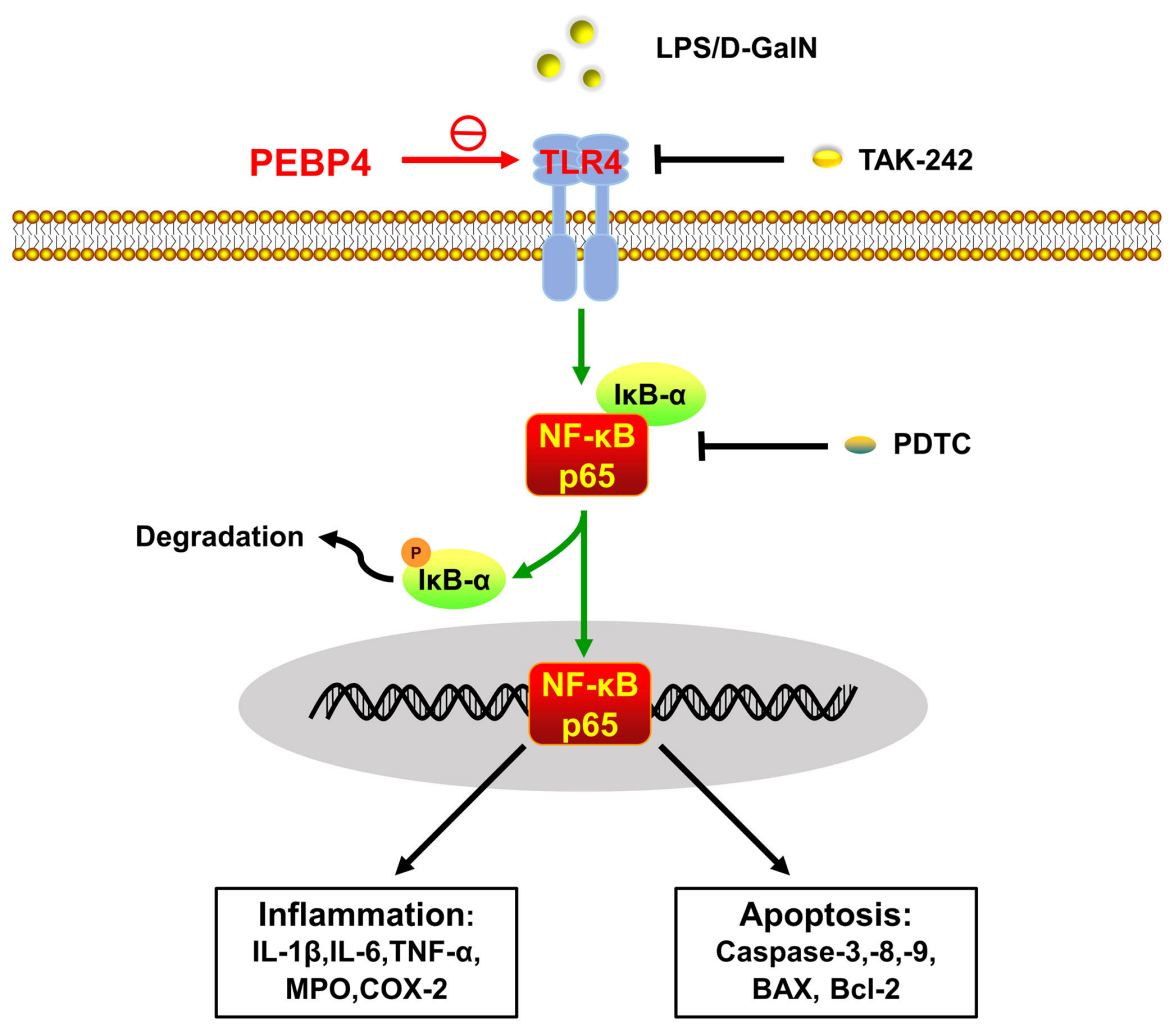
Schematic of PEBP4-driven TLR4/NF-κB signaling pathway-associated inflammatory and apoptotic talk in LPS/D-GalN-induced ALI. PEBP4 CKO could activate the TLR4/NF-κB signaling pathway, promote NF-κB translocation into the nucleus, and then regulate the transcription of inflammatory factors and apoptotic genes to mediate acute liver injury (ALI) from both inflammation and apoptosis. TLR4 inhibitor (TAK-242) and NF-κB inhibitor (PDTC) could partially reverse these effects.

In conclusion, this research demonstrated that PEBP4 CKO aggravated LPS/D-GalN-induced ALI by promoting inflammatory mediators release and apoptosis. Mechanistically, the effect achieves through activation of the TLR4/NF-κB signaling pathway (**Figure 5**).

## Data Availability Statement

The original contributions presented in the study are included in the article/supplementary material. Further inquiries can be directed to the corresponding author.

## Ethics Statement

All animal experiment was approved by the Animal Protection Committee of Jiangxi Medical College of Nanchang University.

## Author Contributions

X-qQ, Q-fC, Q-qS and Q-qL: build model, western blot, RT-PCR, statistical analyses and writing the article. S-yZ, Y-hL, L-yB, and SG: HE staining, TUNEL staining, and Kits assay. Q-fC: modified the article and submitted the article. X-yZ: oversaw the study, designed the experiments, modified the article, and funding acquisition. All authors take responsibility for the manuscript.

## Funding

This work was supported by the National Natural Science Foundation of China (No. 81760117 and No. 81460126), Natural Science Foundation of Jiangxi province (No. 20181BAB205012), National College Students’ innovation and entrepreneurship training program (No. 202010403006), and Jiangxi Students’ innovation and entrepreneurship training program (No.S202010403056).

## Conflict of Interest

The authors declare that the research was conducted in the absence of any commercial or financial relationships that could be construed as a potential conflict of interest.

## Publisher’s Note

All claims expressed in this article are solely those of the authors and do not necessarily represent those of their affiliated organizations, or those of the publisher, the editors and the reviewers. Any product that may be evaluated in this article, or claim that may be made by its manufacturer, is not guaranteed or endorsed by the publisher.
